# Engineering in-vitro stem cell-based vascularized bone models for drug screening and predictive toxicology

**DOI:** 10.1186/s13287-018-0847-8

**Published:** 2018-04-20

**Authors:** Alessandro Pirosa, Riccardo Gottardi, Peter G. Alexander, Rocky S. Tuan

**Affiliations:** 1 Center for Cellular and Molecular Engineering, Department of Orthopaedic Surgery, University of Pittsburgh School of Medicine, 450 Technology Drive, Pittsburgh, PA 15219 USA; 2 Ri.MED Foundation, Via Bandiera 11, Palermo, 90133 Italy

**Keywords:** tissue engineering, vascularized bone, three-dimensional microphysiological systems, biomaterial, scaffold, preclinical model, bioreactors

## Abstract

The production of veritable in-vitro models of bone tissue is essential to understand the biology of bone and its surrounding environment, to analyze the pathogenesis of bone diseases (e.g., osteoporosis, osteoarthritis, osteomyelitis, etc.), to develop effective therapeutic drug screening, and to test potential therapeutic strategies. Dysregulated interactions between vasculature and bone cells are often related to the aforementioned pathologies, underscoring the need for a bone model that contains engineered vasculature. Due to ethical restraints and limited prediction power of animal models, human stem cell-based tissue engineering has gained increasing relevance as a candidate approach to overcome the limitations of animals and to serve as preclinical models for drug testing. Since bone is a highly vascularized tissue, the concomitant development of vasculature and mineralized matrix requires a synergistic interaction between osteogenic and endothelial precursors. A number of experimental approaches have been used to achieve this goal, such as the combination of angiogenic factors and three-dimensional scaffolds, prevascularization strategies, and coculture systems. In this review, we present an overview of the current models and approaches to generate in-vitro stem cell-based vascularized bone, with emphasis on the main challenges of vasculature engineering. These challenges are related to the choice of biomaterials, scaffold fabrication techniques, and cells, as well as the type of culturing conditions required, and specifically the application of dynamic culture systems using bioreactors.

## Background

### Bone architecture and development

**Fig. 1:**
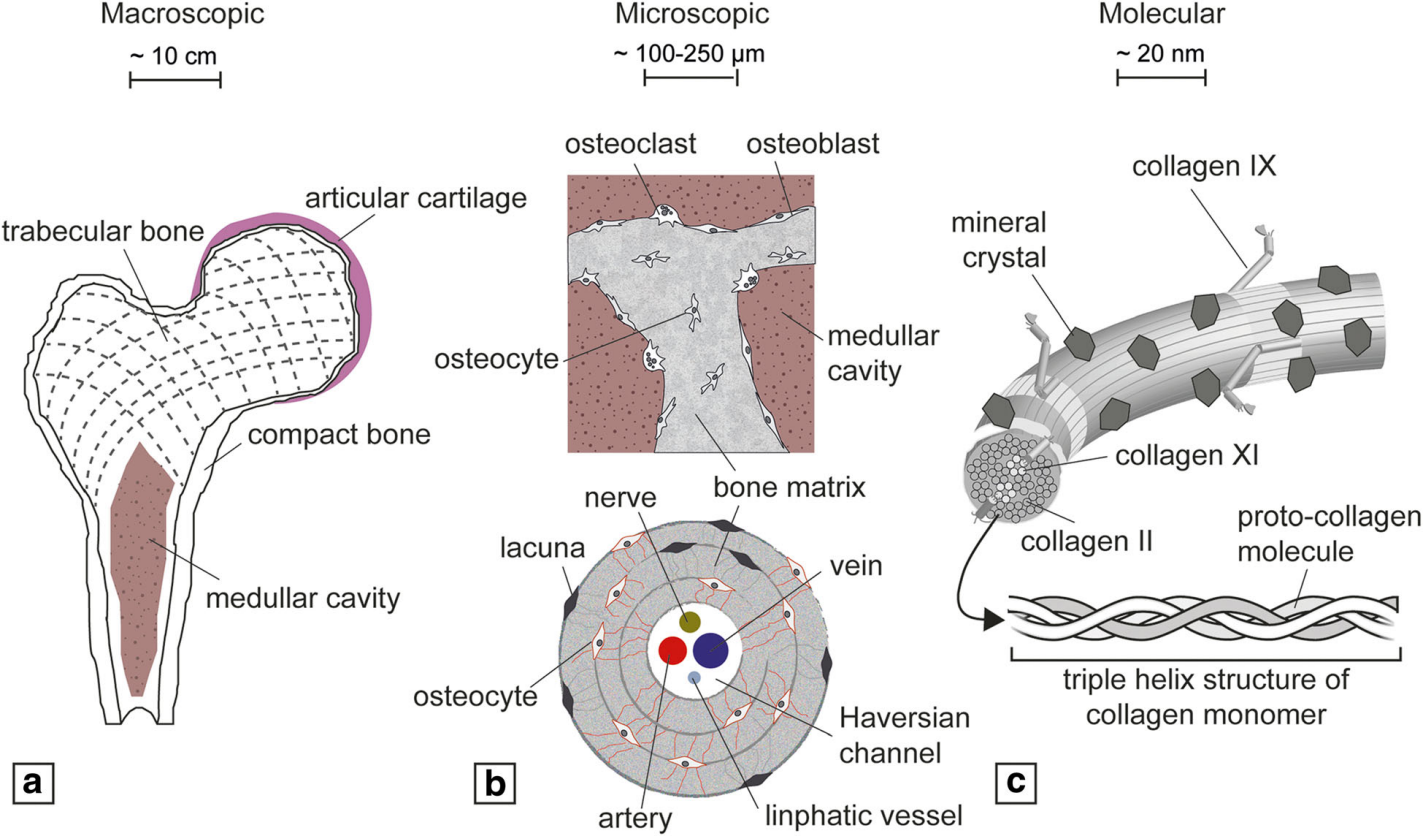
Hierarchic structure of bone and bone tissue from macroscopic (**a**) to microscopic (**b**) to molecular (**c**) levels

Bones are hierarchically organized over multiple length scales. Macroscopically, bone can be divided into compact and trabecular tissues, each with very different mechanical strength and stiffness (Fig. 1a). The microscopic architecture of the former is characterized by osteons and Haversian channels containing nerves and blood supply, whereas the latter is comprised of interconnected trabeculae and the presence of the bone marrow [1] (Fig. 1b). At a molecular level, bone extracellular matrix (ECM) is composed largely of the fibrous macromolecule collagen type I and mineralized inorganic hydroxyapatite crystals [2] (Fig. 1c). Bone is primarily vascularized by an arterial network, but within the bone marrow cavity and the Haversian channels the vasculature branches into thin-walled capillaries, whose fundamental role is the exchange of nutrients and signals between blood and bone cells [3].

Bone fulfills a wide range of physiological functions. As essential structural and load-bearing elements, bones represent the foundation of physical locomotion and protect our internal organs. Due to the presence of the bone marrow, long bones serve as the tissue origin of the biological components required for hematopoiesis. Moreover, osseous tissues can trap potentially harmful metals (e.g., lead), as well as maintain the homeostasis of key electrolytes via calcium and phosphate ion storage [4]. Bone formation can occur through two distinct pathways, intramembranous and endochondral ossification. In both cases, the first step is the condensation of mesenchymal cells to produce a template for subsequent bone formation [5]. Intramembranous bone formation (typical of flat bones) involves the direct differentiation of mesenchymal progenitor cells into osteoblasts, whereas endochondral ossification (typical of long bones) involves the initial differentiation of the mesenchymal progenitor cells into chondrocytes, followed by their hypertrophy, matrix mineralization, and replacement by bone tissue [6]. Both intramembranous and endochondral ossification occur in close proximity to vascular ingrowth. During intramembranous ossification, capillaries invade the differentiating mesenchymal zone, whereas in endochondral ossification, hypertrophic chondrocytes recruit the infiltrating vasculature. Initial vascularization is followed by invasion of osteoclasts and osteoblasts, with coordinated resorption of hypertrophic cartilage and subsequent mineralization of the ECM and bone formation [7]. Hence, vascularization occurs from the ossification centers toward the growth plate and determines the rate of bone ossification [8]. It is interesting to note that many of the processes that occur during long bone formation are recapitulated during fracture healing [9], underscoring the importance of knowledge of bone development for the design of more effective strategies for bone repairing [6]. Vascular endothelial growth factor (VEGF) is a key regulator of angiogenesis and thus bone development [10]. For instance, VEGF-A gene depletion was shown to attenuate the resorption of hypertrophic chondrocytes and bone formation, highlighting the role of VEGF-dependent angiogenesis during bone formation [11]. VEGF-A levels were also found to be dependent on the oxygen-sensing hypoxia-inducible factor (HIF)-1α pathway in the modulation of bone mass, thus confirming the role of neoangiogenesis for the homing of osteoblast progenitor cells and for providing bone formation/promoting factors [12]. In fact, besides bone development, vasculature is also essential for bone remodeling, and fracture healing is also highly dependent on the vasculature. Dysregulated interactions between the vasculature and bone cells are the basis of a number of different pathologies [13]. Some of these, such as avascular necrosis and osteoporosis, are associated with a diminished vascular supply [14, 15], whereas others such as Gorham–Stout disease, a form of idiopathic osteolysis caused by abnormal proliferation of vascular structures originating in the bone [16], and Klippel-Trénaunay syndrome, characterized by vessel malformations and overgrowth of bones and soft tissues [17], are caused by excessive vascularization.

### Bone and related pathologies

Bone diseases can cause loss of bone strength and density, and they may arise from nutrient deficiencies, abnormal development, genetic disorders, impaired vasculature, and other causes. Table 1 summarizes and compares the main pathologies affecting bone.

**Table 1:** Etiology, current treatments, and role of vasculature in the main pathologies affecting bone

	Etiology/risk factors	Current treatments	Role of vasculature
**Osteoporosis**, loss of bone density	Altered balance of bone remodeling: greater bone removal by osteoclasts and then production by osteoblasts [18]	Administration of bisphosphonates, which shorten osteoclast life span and inhibit bone resorption [19]	Possible link between decreased production of vasodilator molecules by endothelial cells and increased bone loss [20]
**Osteoarthritis**, progressive degeneration of cartilage and bone	Traumatic, congenital, postoperative, metabolic, endocrine; age, joint overuse, obesity are common risk factors [274]	Symptomatic treatments through physiotherapy, orthopedic aids and orthoses, pharmacotherapy, total joint replacement [275]	Increased vascularization and neoangiogenesis in the joint; increase in VEGF level in osteoarthritic chondrocytes [43]
**Osteomyelitis**, infection within bone	Infection by *Staphylococcus aureus*, but also by other Gram-negative cocci and Gram-positive bacilli [45]	Parenteral course of broad-spectrum antibiotics and surgical debridement[46]	Poor vascularity can cause both development of the infection and resistance to antibiotics [46]
**Osteonecrosis**, death of bone cells, arthritis, and destruction of bone	Inadequate vascular supply to the bone; long-term steroid treatment, alcohol abuse, joint injury, arthritis, cancer are common risk factors [52]	Nonsteroidal anti-inflammatory drugs to reduce pain and inflammation; bone surgery, grafting, and joint replacement [276]	Compromised subchondral microcirculation, vascular interruption, intravascular occlusion, and extravascular compression [277]
**Fractures**, loss of bone contiguity	Mainly trauma; osteoporosis, low mineral density, age, tumors are common risk factors [278–280]	Fracture reduction and immobilization; bone autograft, allograft, or synthetic materials [59]	Vascular supply is critical for fracture healing; VEGF treatment can enhance fracture repair [57]
**Osteosarcoma**, bone malignancy	Occurring mostly in the medullary cavity of long bones: environmental factors, chromosomal abnormalities, p53 mutation are common risk factors [72]	Depending on the stage, chemotherapy, radiation therapy, surgery (amputation, grafting, local excision) [281]	Vasculature is critical for tumor survival, osteosarcoma generally involves downregulation of anti-angiogenic factors [73, 74]

*VEGF* vascular endothelial growth factor

Osteoporosis refers to the loss of bone density resulting from an altered balance of the bone remodeling process, and affects approximately 10 million US adults 50 years of age and older [18]. The most widely used osteoporosis treatment is the administration of bisphosphonates, which shorten the osteoclast life span and inhibit bone resorption [19]. Although general risk factors of osteoporosis are well documented, little is known about the role of vasculature [20]. Some studies have revealed a connection between low bone mineral density and increased cardiovascular morbidity/mortality [21, 22]. Endothelial cells (ECs) are known regulators of vascular tone by releasing vasodilator molecules, such as nitric oxide (NO), and they have been addressed as a potential link between cardiovascular diseases and osteoporosis. Studies in rats showed that the inhibition of NO production or NO synthase (NOS) activity was followed by marked bone loss [23, 24], while human studies revealed lower NOS expression resulting from estrogen deficiency [25–27]. Since the presence of estrogen receptors has been found in human ECs [28, 29], it is possible that estrogen deficiency seen in postmenopausal women could alter the endothelial function of bone microcirculation. Although these studies suggest that endothelial dysfunction may play a role in the development of osteoporosis, the exact causal relationship has yet to be determined.

Osteoarthritis is the main cause of disability in the USA [30], and its hallmark is the progressive degeneration of cartilage. However, OA affects the whole joint and all tissues play a role in the disease [31]. In particular, the subchondral bone has been reported to be critical in the pathogenesis of OA [32]. During movement, there is continuous functional interaction across the osteochondral junction. Under the diseased state, altered mechanical loading in cartilage induces changes in bone and vice versa [33, 34]. The communication between the two tissues, however, is not limited to mechanical coupling and the associated mechanotransduction. Recent evidence indicates that the calcified cartilage and subchondral bone are not an impermeable barrier, and some molecules are capable of diffusing across the osteochondral junction [35–38]. Blood vessels and microchannels have been found to reach from the subchondral bone all the way to the uncalcified cartilage, and there is evidence of contact between uncalcified cartilage and subchondral bone and the marrow spaces [33, 39–41]. During OA, the osteochondral junction is significantly altered, allowing greater transport and cellular crosstalk between cartilage and bone [32, 38, 42]. Another hallmark change of the osteochondral junction occurring during OA is increased vascularization and neoangiogenesis [38, 43], which may further contribute to the molecular crosstalk between cartilage and bone. Part of this signaling involves an increase in the VEGF level in osteoarthritic chondrocytes compared to those in healthy cartilage [43], possibly contributing to the induction of vascular invasion as part of a proregenerative mechanism. In turn, ECs have recently been reported to enhance chondrogenic differentiation of mesenchymal stem cells (MSCs) [44], suggesting the potential of significant molecular interplay between subchondral bone vasculature and cartilage, an aspect that has not been much investigated. Overall, increased vascularity in the subchondral bone is associated with OA severity in cartilage and with clinical disease activity [33].

Another pathogenic bone condition with devastating consequences is osteomyelitis (OM). OM can be broadly defined as an infection within the bone and is classified by duration (acute or chronic), pathogenesis (trauma, contiguous spread, hematogeneous, surgical), site, extent, or type of patient [45]. Poor vascularity is a prime cause for both the development of an infection and resistance to antibiotics [46]. Acute OM can be eradicated before osteonecrosis occurs if the infection is treated promptly and aggressively with antibiotics and surgical debridement [46]. However, in an established/chronic infection, fibrous tissue and chronic inflammatory cells encapsulate the infected site, reducing vascular supply, inhibiting an effective inflammatory response, and limiting the action of antibiotics [46]. In addition, the bacteria become encapsulated within a biofilm, that both protects the bacteria from the body’s defenses and antibiotics and serves as a chronic source of new bacterial infection [45–47]. Several therapies intended to enhance blood flow to vascular or chronically infected areas have been tested in experimental models or clinical trials, including hyperbaric oxygen therapy, PRP, and VEGF delivery [47–49]. Recently, a VEGF gene-transfected muscle flap was shown to be effective as a treatment to supplement systemic antibiotic treatment in the management of experimental OM in a rodent model [50, 51]. These strategies seek to enhance the body’s own defense and the effectiveness of antibiotics by enhancing blood flow to avascular tissue.

Osteonecrosis, or avascular necrosis (AVN), occurs when the blood supply to the bone is disrupted, precipitating death of the bone cells, arthritis, and destruction of the hip joint. Common risk factors include long-term steroid treatment, alcohol, abuse, joint injury, arthritis, and cancer—all associated with altered blood supply [52]. The risk greatly increases with corticosteroid use (to treat pain and inflammation), bisphosphonate (to prevent bone loss), and anti-angiogenic therapy (in the treatment of some cancers or leprosy) [53, 54]. Areas susceptible to AVN have several common characteristics: they have limited routes of vascular supply, they undergo relatively high rates of bone turnover, and they may have higher than normal exposure to bacterial infections [55]. Evidence that reduced vascular supply causes AVN is indicated by the rise in AVN incidence following anti-angiogenic treatments used in cancer therapy, such as the VEGF-specific antibody bevacizumab, the tyrosine kinase inhibitor sunitinib (Sutent), and the mTOR inhibitor rapamycin, and new treatments for leprosy, such as lenalidomide. Osteoclasts and blood vessels are closely associated during bone remodeling, and recent studies indicate that osteoclasts promote angiogenesis through secretion of factors such as matrix metalloproteinase 9 (MMP9). Poor vascularization occurs in certain inflammatory and immunosuppressive states and in the presence of infection as well. While the pathogenesis of AVN is largely agreed upon, the cellular and molecular mechanisms are less understood, and thus counter-treatments to prevent the condition during anti-resorptive and anti-cancer treatments are not available.

Trauma-related injuries can lead to bone fractures, whose healing is critically dependent upon an adequate vascular supply. Breakage of the bone initiates the fracture healing process, which begins with an inflammatory reaction, followed by the processes typical of endochondral ossification for a period of up to 28 days. Neovascularization is critical for successful bone formation, since vascular endothelium interruption is the first event following trauma, which could lead to the formation of necrotic tissue. Experimental evidence showed impaired bone formation with administration of anti-angiogenic factors [56], whereas VEGF treatment enhanced fracture repair [57]. Clinical evidence showed that in the presence of decreased vascular perfusion to the fracture, the incidence of impaired healing (delayed union or nonunion) increases from 10–15% to 46% [58]. Standard treatment to augment bone healing and prevent delayed union or nonunion is represented by the use of autograft, allograft, or synthetic materials [59]. Bone marrow-derived endothelial progenitor cells (EPCs) participate in the generation of new blood vessels (vasculogenesis) at the site of injury [60, 61], and they have been shown, in combination with bone marrow-derived MSCs, to augment bone healing [62, 63]. Significant progress in improving fracture healing is hindered by the limited knowledge of the molecular mechanisms leading to nonunions; thus, a better understating of the biological pathways involved in this process would certainly benefit from the development of specific clinical therapies.

Bone also plays a role in pathologies related to ionic homeostasis, such as calcium and phosphate [64]. In particular, osteocytes are involved in phosphate homeostasis through the expression of different proteins, such as dentin matrix protein 1 (DMP1) [65], Phex, and fibroblast growth factor 23 (FGF-23) [66]. In addition to being key ions in systemic physiology, including muscle functions, calcium and phosphorous homeostasis is crucial for the development of the growth plate, which must be mineralized to promote proper vascular invasion and subsequent bone formation [67]. Bone may also act as a reservoir of lead, contributing to systemic toxicity [68], and it is the target tissue for the actions of a number of teratogens (e.g., valproic acid, thalidomide, etc.) [69, 70]. Studying the mechanism of how these toxins act at the cellular and molecular levels with bone is indeed crucial to developing new treatments.

Osteosarcoma is the most common bone malignancy affecting predominantly adolescents and young adults, with a 5-year survival rate of about 50–60% [71]. Osteosarcoma occurs mostly in the medullary cavity within the metaphysis of long bones, an active bone growing region, and then can propagate to the bone cortex and the surrounding soft tissues. The molecular pathogenesis of this tumor is quite complex and involves several elements, such as environmental factors (e.g., UV and ionizing radiation, methylcholanthrene and chromium salts, etc.), chromosomal abnormalities, p53 mutations, and so forth [72]. As for many other tumors, vasculature is a critical factor for the survival and proliferation of cancer cells; in fact, osteosarcoma generally involves downregulation of anti-angiogenic factors, such as thrombospondin-1 [73] and pigment epithelium-derived factor (PEDF) [74]. Although osteosarcoma is known as a vascular tumor, there are contradictory data about the correlation between the microvascular density/VEGF expression and the formation of metastasis [75, 76]. Emerging evidence suggests that the blood vessels of bone may play a role in the interactions between other tumors and the bone microenvironment in the pathogenesis of bone metastasis [77]. The extravasation of tumor cells can be related to the molecular receptors typical of a tissue/organ-specific blood vessel [78]. Interestingly, blood vessels located in the metaphysics of long bones express specific adhesion proteins, such as P-selectin and E-selectin, which have been shown to promote interaction and subsequent adhesion of tumor cells [79]. One widely studied example of this kind of interaction is the preferential spreading of breast cancer metastasis to bone. In fact, breast cancer cells express chemokine receptors, integrins, cadherins, and bone-remodeling factors that contribute to the successful and preferential spread of tumor to bone [79].

Due to the multifactorial nature of bone diseases, the aging population, and increased occurrence of trauma-related injuries, bone disorders represent a big concern and the current treatments do not provide optimal outcomes. Furthermore, any alteration in the vascular supply may lead to an increased susceptibility to osteoporosis, osteonecrosis, and osteomyelitis [8]. Animal models have been traditionally utilized in research to mimic these human pathologies. Nevertheless, on multiple occasions, animal models have been unsuccessful and unreliable in predicting the processes and development of human pathologies and the response to drug candidates, because their physiology is fundamentally different from that of humans. To overcome this deficiency, tissue engineering may represent an alternative platform by establishing in-vitro pathophysiological models based on human cells to study the causes and progression of specific diseases, and to develop and test candidate therapeutic strategies.

## In-vitro modeling of 3D vascularized bone models through tissue engineering approaches

The successful development of in-vitro engineered bone is critically dependent on the ability to introduce a 3D vascular network that guarantees adequate oxygenation, mass transfer, nutrient delivery, and by-product removal [80]. Here, we summarize the challenges in generating in-vitro vascularized bone constructs and the main materials, scaffolds, and cells required to achieve this goal, with a focus on stem cell employment.

### Challenges of in-vitro vascularized bone engineering

**Fig. 2:**
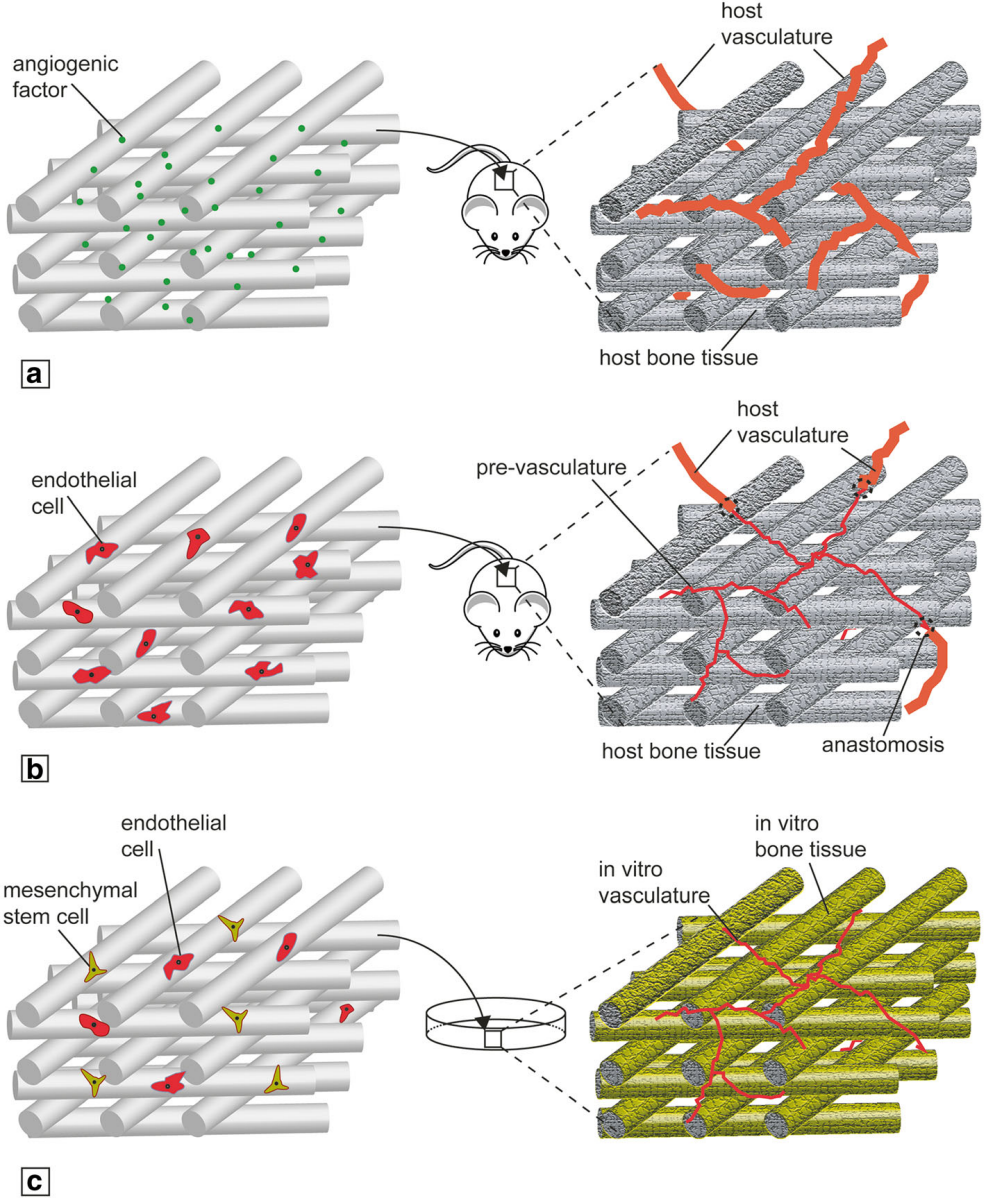
Vascularization strategies for engineered bone constructs. **a** Scaffold loading with proangiogenic factors in-vivo. **b** Scaffold preseeding with ECs in-vivo. **c** Coculture strategy more suitable for in-vitro studies

Bone is a highly vascularized organ and the development of vasculature and mineralized matrix requires a synergistic interaction between osteogenic and endothelial precursors [81]. These mechanisms have been studied in animal models, but they are still not understood due to the complexity of the in-vivo environment. In-vitro scale-up of bioengineered tissues is known to be limited by diffusion issues; therefore, the establishment of a functional vasculature within the construct could be essential to generate an accurate and large enough model capable of mimicking the native tissue. Current vascularization strategies comprise the use of angiogenic factors combined with 3D scaffolds (Fig. 2a), prevascularization strategies (Fig. 2b), and the use of coculture systems (Fig. 2c) [82, 83].

The first approach relies on the induction of vascularization by endothelial precursors using angiogenic factors such as VEGF. For instance, Braghirolli et al. [84] demonstrated that an electrospun poly(caprolactone) scaffold loaded with VEGF promoted the penetration and proliferation of EPCs within the 3D matrix. This process is primarily EC regulated and, in the context of TE, specific interactions with the scaffold material and other cell types are needed for optimal in-vitro vascularization. Thus, the current research trend is focused on the production of prevascularized constructs in-vitro. This approach proposes the generation of stable vasculature in-vitro using ECs and subsequent in-vivo implantation in the target site [85]. The principal drawback of this method, when utilized for in-vivo bone repair, is the difficulty of generating stable in-vitro vasculature prior to implantation. The use of coculture systems including different cell types is more complex and several parameters must be taken into account for the successful outcome of a vascularized bone construct, namely cell type (osteogenic and vasculogenic), media, seeding methodology, culture (static or dynamic), scaffolds, and microenvironment (e.g., oxygen tension) [83]. In the past decade, different attempts to generate vascularized bone constructs using osteogenic precursor cells and vascular progenitor cells have been made, but the majority of these studies were conducted through in-vivo models [86–88]. It has been difficult to create a veritable in-vitro model containing bone tissue and vasculature-like structures within the same construct. A recent attempt to introduce simultaneously osteogenic differentiation and vasculature development in-vitro was made by Tsigkou et al. [89], which combined human bone marrow MSCs and human umbilical vein endothelial cells (HUVECs) seeded on a polymeric scaffold and in a hydrogel, respectively. They observed the formation of capillary-like structures 4–7 days after implantation in a mouse model, and MSCs were found to be necessary for the development of a stable vasculature. The main outstanding issue is still the generation of accurate in-vitro models, due to the difficulties of obtaining quality mineralized bone matrix, further complicated by the introduction of the vasculature.

Bone mineralized matrix formation is provided by osteoblasts during embryonic development and postnatal growth, yet about 95% of the cellular population residing in the adult skeleton is represented by osteocytes. Osteocytes are the terminally differentiated osteoblasts embedded in the lacunae within the bone matrix. They are characterized by cellular processes radially spread toward the mineralized matrix inside tiny tunnels called canaliculi, filled by canalicular fluid [90]. The presence of osteocytes in an engineered bone construct is an indication of a mature developed osseous tissue. Although osteocytes play a secondary role in bone tissue formation, they are functionally important in its homeostasis, mechanosensation, and mechanotransduction [91, 92]. Osteocytes sense canalicular fluid flow-derived shear stress through their primary cilium [92] and specific ion channels and mechanotransduction proteins, releasing in response paracrine signaling molecules such as NO, ATP, and prostaglandins, which regulate osteoblast and osteoclast activity [93–95]. The lacuna–canalicular network connects the osteocytes to the vasculature and there is evidence linking osteocyte apoptosis and reduced VEGF production with consequent impaired vascularity and bone strength [96]. Taken together, these findings highlight the importance of the presence of mature osteocytes in an engineered bone. For this reason, many investigations are focused on the derivation of mature osteocytes from stem cells to better understand their physiology and assess their potential in bone engineering [97–99].

Bone is also innervated by sensory and sympathetic nerve fibers that, in addition to skeletal pain transmission, play a role in bone metabolism. Bone cells have been recently shown to express adrenoceptors, receptors for norepinephrine (NE), calcitonin gene-related peptide (CGRP), substance P, and vasoactive intestinal peptide (VIP) [100, 101]. NE reuptake influences bone remodeling by osteoclasts [102], CGRP deficiency was linked to decreased bone formation in mice [102], while SP has been shown to have both dose-dependent bone-resorbing and formation activity [102]. These are just a handful of examples on how innervation has been linked to bone production and turnover; thus, an in-vitro engineered bone construct would certainly benefit from the presence of nerve endings and related neurotransmitters. However, the generation of a vascularized and innervated bone construct clearly presents an additional level of complexity in terms of integrating and regulating such different tissue types [103].

Another parameter to consider when developing veritable in-vitro vascularized bone is the incorporation of osteoclast activity inside the engineered constructs. Osteoclasts are essential for bone remodeling by resorbing bone matrix and generating physical space for osteoblasts and ECs, thus allowing the formation of new bone tissue and vasculature [104]. The dissolution of the inorganic phase of bone matrix takes place by the secretion of hydrochloric acid, while the organic matrix is resorbed by secreted enzymes, like cathepsin K and metalloproteinase 9 [105]. The resorption activity as well as the area and depth of the resorption pits can be analyzed in-vitro using osteoclast-like cells derived from monocytes, for instance the RAW 264.7 cell line [106]; however, to date, there are considerable variations in the examined parameters. There is no specific, single functional osteoclast cell marker; thus, different parameters should be analyzed together, such as tartrate-resistant acid phosphatase 5b (TRAP 5b) [107], morphological changes from monocytes [104], and 3D volume characterization of pits [108]. Furthermore, as different materials can affect the response of osteoclasts, the choice of the scaffold is critical for an optimized bone engineering strategy. Different authors have studied the resorption ability of osteoclasts using different scaffolds/materials, from dentin to bone substitutes, as well as calcium phosphate and bioactive glasses [109–112], and have observed specific resorption rates depending on the material used. For example, Keller et al. [112] found that natural biomaterials were resorbed more rapidly than synthetic ones, while Badran et al. [111] showed an inhibition of osteoclast activity by increasing mineral density.

As highlighted by Alexander et al. in a recent minireview [31], a TE approach can be used for the development of in-vitro preclinical models of normal/pathological tissue function, but an effective high-throughput assay should consist of a minimal system with well-defined performance parameters. These systems should model the structure and function of human tissue, as well as the physiological response to different stimuli, such as the interaction with adjacent tissues [113–115]. Bone actively interacts with cartilage, muscles, ligaments, tendons, and many other tissues in its physiological function within the musculoskeletal system [116–120]. An ideal in-vitro model of bone should consider the possibility of studying its interaction with other tissues, thus posing significant challenges in accommodating different, tissue-specific microenvironments.

Due to their pluripotency/multipotency, stem cells represent an exceptional tool to achieve this complex integration between bone and other tissues. A good model to study the interaction between bone and cartilage was developed by Lin et al. [121], who produced a biphasic construct mimicking the nature of the osteochondral junction. This construct was developed using a 3D-printed dual-chamber bioreactor that allowed the creation of two separate yet communicating microenvironments [122]. Osseous and chondral phases were generated using one stem cell type, bone marrow-derived MSCs, encapsulated in a photocrosslinked gelatin methacrylate (gelMA) hydrogel. Osteogenic and chondrogenic media were used to differentiate MSCs toward bone and cartilage phenotype, respectively. The two constructs were independently controlled and tested by the introduction of bioactive agents or candidate drugs.

**Fig. 3:**
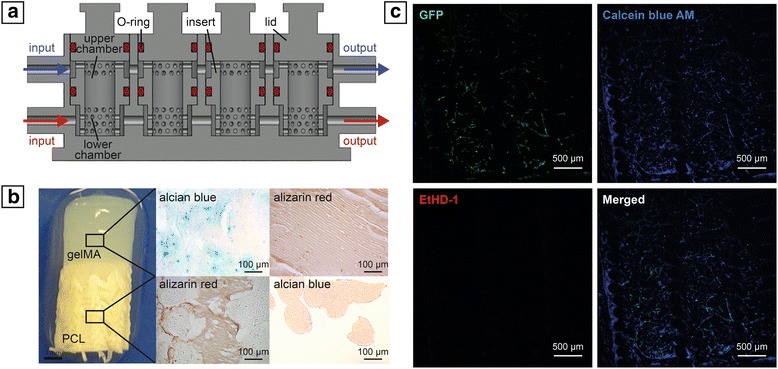
**a** Cross-sectional bioreactor schematic. **b** Macroscopic and histological analysis of engineered osteochondral interface. **c** Live/dead staining of capillary-like network formed by HUVECs in bone compartment (GFP, green = HUVEC; Calcein Blue AM, blue = live cells; EthD-1, red = dead cells). gelMA gelatin methacrylate, PCL poly(ε-caprolactone), GFP green fluorescent protein, EthD-1 ethidium homodimer-1

Although different models have been created to recapitulate the interaction between bone and other adjacent tissues, to the authors’ knowledge, an in-vitro engineered model coupling vascularized bone to another tissue has not yet been developed. Such capability would be particularly relevant when engineering the osteochondral junction, as only the simultaneous presence of bone matrix, vasculature, and cartilage can enhance or permit veritable behavior of the complex. In fact, the interactions between vasculature and cartilage are well known during in-vivo skeletogenesis [123, 124], and numerous in-vitro studies have documented the molecular exchanges driving the inhibition of chondrocyte differentiation, including the pivotal role exerted by VEGF secreted by ECs [125–127]. However, a model also containing subchondral bone would better mimic the complex interaction among the three tissues. For these reasons, a triphasic scaffold was produced by the authors using a recently developed 3D-printed microphysiological tissue system (MPS) bioreactor that allows the separate flow of specific media to the chondral and osseous components, while maintaining them in contact and allowing tissue–tissue communication [128] (Fig. 3). The cartilage construct was engineered incorporating human bone marrow-derived MSCs in a photocrosslinkable gelMA hydrogel, while the vascularized osseous construct was obtained by seeding MSCs and HUVECs in a poly(ε-caprolactone) (PCL) scaffold, produced through additive manufacturing as described by Puppi et al. [129]. Results from our preliminary assessments suggest a better differentiation of MSCs in the presence of the HUVECs, which form interconnected tubular-like structures in the osseous compartment.

Overall, the development of a successful in-vitro model of vascularized bone is heavily dependent on optimal selection of scaffold/matrix able to guide the formation of the different tissues, and on fine control of cell signaling.

### Materials

Mineralized bone and associated vasculature are characterized by different morphological and structural properties, thus different biomaterials are needed for the engineering of both tissues to match their characteristics. Different materials have been investigated for the development of bone constructs, and evaluated both in-vitro and in-vivo. Calcium phosphates (CaPs) have been of great interest due to their osteoconductive properties. The majority of research has been focused on two main forms of CaPs, hydroxyapatite (HAp) and beta-tricalcium phosphate (β-TCP), or the combination of both [130]. Other inorganic materials that have been exploited in bone engineering are bioactive glasses [131], which have been shown to improve the formation of a hydroxycarbonate apatite layer by osteoblasts in-vitro and to support bone formation in-vivo [132]. The most commonly used polymeric materials for bone engineering include many natural polymers, such as collagen, fibrin, alginate, silk, hyaluronic acid, chitosan, and polyhydroxyalkanoates [133]. These biopolymers offer economic and environmental advantages, such as low manufacture/disposal costs and renewability, as well as biological advantages, such as supporting cell signaling, adhesion, and cell-mediated degradation and remodeling [134]. Synthetic polymers have also been thoroughly investigated as engineered bone scaffolds, by virtue of their controllable and reproducible chemical–physical and degradation properties, which can be tailored to the requirements of the target applications [135]. The different classes of polymers that have received significant attention include saturated poly(α-hydroxesters), such as poly(glycolic acid) (PGA), poly(lactic acid) (PLA), and PCL, and biodegradable polyurethanes, covering a wide range of properties, including mechanical strength, elasticity, biodegradability, hydrophilicity, and so forth [135]. When considering the development of a capillary-like network within a mineralized bone construct, the most current models include the embedding of endothelial-derived cells in hydrogels, such as Matrigel^®^, collagen, and fibronectin [136–138]. However, these materials have limitations in mechanical stability, durability, and immunogenicity when implanted [139, 140]. One recent example to overcome these limitations is represented by the use of methacrylated gelatin (gelMA), produced by conjugating methacrylate groups to the amine-containing side groups of gelatin, which becomes a photocrosslinkable hydrogel [141]. This material is characterized by the advantages of both natural and synthetic polymers, allowing the fine-tuning of mechanical properties, while preserving biological cues. Different studies in the past have reported the capability of creating vascular-like structures using gelMA and ECs [80, 89, 142].

There have been a number of investigations that explored a variety of biomaterials for the engineering of vascularized bone; however, identifying the most suitable biomaterials remains a difficult task, since each material has its inherent drawbacks. Ceramics are characterized by brittleness and the biodegradation rate not matching the formation of new bone tissue; natural polymers possess low mechanical, thermal, and chemical stability; and synthetic polymers lack biological cues. A logical path to overcome these limitations would be to combine different materials to obtain constructs with improved properties. An example of this paradigm is the development of nanocomposite materials based on biopolymers and ceramic nanofillers, in an attempt to exploit the biological activity of natural polymers as well as the osteoconductivity of ceramics [143].

### Scaffolds

All of the biomaterials already described require multistep processing to develop 3D scaffolds/matrices capable of inducing and supporting osteoblast proliferation and differentiation, as well as vasculature development. Accordingly, in addition to osteoinductivity and osteoconductivity, an ideal scaffold should have functional physical properties such as interconnected macroporosity with pore size larger than 100 μm for optimal osteoblast differentiation and formation of new blood vessels, and biocompatible stiffness to match the mechanical properties of native bone [144]. Substrate mechanical properties greatly influence stem cell osteogenic differentiation, as well as EC behavior [145]; for instance, a scaffold Young’s modulus in the range 25–40 kPa directs MSCs toward osteoblastic differentiation [146, 147]. Current technologies do not allow the production of scaffolds with the exact mechanical properties of bone; however, a number of groups have explored development-inspired precursor templates to instruct stem cells to form a mature tissue [148–151]. Although such constructs do not possess the same mechanical properties as native bone initially, they are engineered in-vitro with sufficient stiffness to be implanted in a load-bearing region and are able to guide stem cells to develop mature bone. For instance, Daly et al. [152] produced an MSC-laden 3D-printed hypertrophic cartilage template, made of a reinforced alginate bioink, which developed into functional vascularized bone when implanted in mice.

Biologically inspired scaffolds should also harbor signals that act to induce the simultaneous development of bone and vasculature. Different biological scaffolds have been used for the development of in-vitro vascularized bone models, including decellularized bone and specific ECM preparations. Decellularized bone has been historically considered and applied as a biologically derived matrix, able to induce mineralized bone production by osteoprogenitor cells. A recent study by Correia et al. [81] showed the employment of decellularized bone plugs as scaffold for HUVECs and MSCs seeded in a fibrin carrier. Vasculature was able to grow inside the porosity of the scaffold both in-vitro and then in- vivo after subcutaneous implantation in mice. The use of decellularized ECM has been reported recently by Gao et al., who developed a vascular patch using MSCs in a decellularized human aortic matrix [153]. Another strategy involves the functionalization of “smart” scaffolds with angiogenic and osteogenic factors such as VEGF, providing a highly localized signal to control stem cell fate [154].

The need to control macrostructural and microstructural properties of a scaffold to meet key requirements of a specific application has led to the development of different manufacturing technologies, such as solvent casting/particulate leaching, freeze drying, phase separation, and combinations of these techniques [135], each conferring to the scaffold different properties. For instance, using a thermally induced phase separation (TIPS) technique, Mannella et al. [155] developed a porous scaffold with a pore size gradient able to mimic the porosity of the cancellous bone. Although several studies about the production of bone scaffolds using the aforementioned techniques have been published, these methodologies are lacking in control over the fine architecture of the structure, specifically in relation to pore morphology and interconnection, fundamental requirements for the introduction of vasculature. There are no precise values for specific bone and/or vascular ingrowth; pore sizes greater than 100 μm have been shown to favor bone formation [156], while capillary density is promoted when pore sizes are greater than 300 μm [130]. For these reasons, solid freeform fabrication (SFF) or additive manufacturing (AM) techniques are attracting great interest, owing to their capabilities of producing predefined interconnected porous structures, using natural and synthetic polymers, as well as ceramics as starting materials [157]. AM techniques comprise the layer-by-layer building of 3D structures, based on computer-aided design (CAD) and computer-aided manufacturing (CAM) processes. Depending on the specific working principles, AM can be classified into laser-based systems (e.g., selective laser sintering, stereolithography), printing-based systems (e.g., 3D printing), and nozzle/extrusion-based systems (e.g., fused deposition modeling, computer-aided wet-spinning), each allowing for specific scaffold macroarchitectural and microarchitectural features [158]. AM has significantly improved the technical ability to control key factors in bone scaffolds, such as composition, pore geometry, size, and interconnectivity, as well as scaffold mechanical performance. The fine-tuning of morphological parameters provided by AM allowed researchers to produce scaffolds for the concomitant development of osseous and vascular tissue. 3D printing has been thoroughly exploited for the generation of bone scaffolds with vascular integration, mostly using ceramics [159], but also synthetic polymers [160], natural polymers [160], and composites [160]. 3D-printed scaffolds have also been loaded with bioactive molecules, such as BMP and VEGF, to promote bone formation and vascularization respectively. Novel studies are exploring the incorporation of other molecules, such as KR-34893 indene compound that stimulates MSC differentiation and mineral deposition [160], oxygen-releasing agents like calcium peroxide to solve O_2_ diffusion issues [160], and platelet-rich fibrin to stimulate bone marrow-derived MSC differentiation [160]. AM and 3D-printing technologies have also produced great advancements in the field of microfluidics systems and organ-tissue chips that employ stem cells for in-vitro disease modeling and drug screening [161–163]. In-vitro vascularized bone models have steadily benefited from this technology. For instance, Jusoh et al. [164] recently reported a microfluidics platform based on HAp-loaded ECM to study angiogenesis and osteogenesis in a vascularized bone model. Furthermore, AM can be integrated with other scaffold fabrication methods to fabricate hybrid architectures with unique structural features, as described in detail by Giannitelli et al. [165]. Although AM techniques were widely studied for the development of bone scaffolds, to date, there are few studies employing them to develop in-vitro models of vascularized bone. A summary of the main scaffolds and fabrication techniques employed in bone engineering is presented in Table 2.

**Table 2:** Major scaffolds and fabrication technologies used in bone engineering

Type of scaffold			Fabrication techniques		
Biologically inspired	Decellularized bone [282–284]	Pros: mimicking bone microenvironment; interconnected porosity for vasculature introduction; osteoinduction and osteoconduction; biomechanical properties	Conventional techniques	Solvent casting/particulate leaching, gas foaming [285, 286]	Pros: ability to generate interconnected porous scaffolds; porosity and pore size can be controlled by altering particle concentration and size or gas concentration
		Cons: difficulty to obtain clinically relevant volumes; specialized perfused apparatus for decellularization; challenge of generating specific anatomical shapes			Cons: inability to produce thick constructs; pore shape and interconnection cannot be controlled
	Extracellular matrix [287–289]	Pros: promote the migration and proliferation of progenitor cells; provide molecules for cell–matrix interactions; provide a structure for mechanotransduction signals		Phase separation [285, 286]	Pros: incorporation of biomolecules within the structure due to mild processing conditions; scaffold customization by altering material and concentration, phase transitions, and/or solvents
		Cons: challenge to minimally disturb biochemical and mechanical properties of the ECM during decellularization; inhomogeneous distribution during cell seeding			Cons: limited material selection and inadequate resolution
Natural/synthetic materials-based scaffolds	Natural polymers [134]	Pros: inherent biocompatibility and bioactivity; can be modified to provide a wide variety of original features; renewability	Additive manufacturing	Selective laser sintering, 3D printing [290, 291]	Pros: control over scaffold internal and external morphology; high production rate; ability to produce large-size scaffolds
		Cons: insufficient mechanical properties; challenge in generating specific morphologies due to poor processing conditions			Cons: laser intensity can induce scaffold degradation; generally low mechanical properties; limited and high-cost materials; high roughness of scaffold’s surface; trapped material inside the scaffold
	Natural ceramics (β-TCP, HA, bioactive glass) [292–294]	Pros: capability to form direct bonds with living bone; osteoinduction and osteoconduction		Fused deposition modeling, computer-aided wet-spinning [157, 158, 165]	Pros: control over scaffold internal and external morphology, pore size, distribution, and interconnection; good mechanical properties; no material trapped in the scaffold
		Cons: brittleness, difficulty of shaping			
	Synthetic polymers [135, 295]	Pros: high versatility regarding control over physical–chemical properties and morphology; easy processability; batch-to-batch reproducibility			Cons: relative regular structures; resolution dependent on the utilized machine
		Cons: lack of important biomolecules aiding cell attachment; may degrade into unfavorable products, such as acids	Bioprinting [296, 297]		Pros: geometry and dimension of the cell-laden construct can be controlled by automated process; nonelevated temperatures required
					Cons: careful attention to cell viability, densities, and ratios during and after printing; printability of the selected bioink material

*ECM* extracellular matrix, *TCP* tricalcium phosphate, *HA* hydroxyapatite

Another fabrication technology that is increasingly gaining attention is bioprinting, which refers to the predefined and precise dispense of cell-laden biomaterials for the construction of complex “living” 3D structures [166]. If the cell selection is represented by osteoprogenitor stem cells, the resulting structure will be composed of cells capable of producing bone matrix. The geometry and dimension of the construct can be controlled in an automated manner such that specific morphologies may be fabricated based on the application. Bioprinting, thus, represents another promising approach for the production of in-vitro models of bone [167]. Traditionally, bioprinting has been applied mainly to the formation of soft tissues and in-vitro vasculature. For instance, Norotte et al. [168] bioprinted single-layered and double-layered vascular tubes with a diameter ranging from 0.9 to 2.5 mm, using different vascular cell types, such as smooth muscle cells and fibroblasts. The identification of new stem cell sources as vascular precursors, coupled with the advancement in bioink and microfluidics technologies, has allowed bioprinting to create constructs that mimic the arrangement of the vasculature in bone for more precise organ modeling. Numerous studies of vasculature bioprinting and chips using stem cells have disclosed different aspects of blood vessel biology, such as the role of transforming growth factor beta on normal vascular function [169], the role of MSCs in promoting vasculature formation [170], regulation of the perivascular stem cell niche by MSCs [171], and the role of angiopoietin in MSC transition to mural cells in the presence of ECs [172]. Bone bioprinting requires the employment of materials with better mechanical properties, needed to mimic the stiffness of the bone matrix. One example of mechanically reinforced bioprinted construct has been made from alginate and PLA nanofibers, supporting adipose-derived MSC viability and differentiation [173]. Another approach is the use of stem cell-laden hydrogels in combination with thermoplastic fibers made of PCL or poly(vinyl alcohol) (PVA) [174, 175]. Gao et al. [176] developed a photopolymerized acrylated peptide, coprinted with poly(ethylene glycol) (PEG) which showed robust osteogenesis of MSCs. MSC osteogenesis has been also stimulated by combining low-intensity pulsed ultrasound (LIPUS), as mechanical stimulation, and a 3D-bioprinted PEG–RGD construct [177]. Other materials have been employed, in various formulations, for the development of bioprinted bone constructs, ranging from agarose and gelatin to PEG [178–180]. The integration of bioprinted vasculature and mechanically stiffer substrates would greatly improve the production of functional in-vitro vascularized bone. One example of this paradigm featured a dual 3D bioprinting system based on fused deposition modeling (FDM) and selective laser ablation (SLA) [181]. The authors used alternate deposition of stiff polylactide (PLA) fibers and MSC/HUVEC-laden gelMA hydrogel to achieve proper mechanical strength and biological cues of complex vascularized bone constructs. Another approach comprised the production of a mandible fragment using an integrated tissue-organ printer (ITOP), which was able to bioprint Pluronic-F127 hydrogel laden with human amniotic fluid-derived stem cells (hAFSCs), in combination with a PCL-based mechanical backbone, and featuring incorporated microchannels for nutrient diffusion [182]. Kolesky et al. bioprinted a thick vascularized tissue using different polymeric templates and fugitive inks. The construct was laden with MSCs in combination with HUVECs, as well as other parenchymal and stromal cells, showing robust osteogenesis and functional perfusion of the vasculature [183].

The great landscape of current scaffold fabrication technologies has provided a wealth of choices for the generation of in-vitro vascularized bone constructs. Future attention should be focused on high-throughput and high-resolution AM techniques that are able to produce functional vasculature within a mature bone construct. The key aspect is the biocompatibility of these methods with the chosen cells of interest in order to obtain their optimal functionality.

### Osteogenic and vascular precursors

When considering the development of an in-vitro model of vascularized bone, the selection of suitable cell sources is crucial. A desired cell source must have little to no limitation in terms of availability and be easy to maintain and manipulate in-vitro. Adult MSCs are widely employed for somatic TE by virtue of their ability to differentiate into multiple lineages, such as cartilage, fat, muscle, and bone [184]. MSCs can be readily isolated from different tissue sources, such as adipose, muscle, bone, and in particular, bone marrow, which is the most widely used source [185]. In fact, bone marrow-derived MSCs have been benchmarked as one of the most appropriate cell sources for bone TE due to their well-defined osteogenic differentiation [186–188]. By using an appropriate culture medium, MSCs can be expanded without differentiation, and induced to undergo specific differentiation to a stable phenotype in culture [189]. In addition to bone marrow-derived MSCs, adipose-derived MSCs (ASCs) are now a widely accepted source for bone tissue engineering applications and have been employed also in numerous preclinical and clinical studies [190]. Induced pluripotent stem cells (iPSCs) and embryonic stem cells (ESCs) have also been studied as potential cell sources for bone regeneration [191–194]. Although pluripotent ESCs are a promising cell candidate for the development of fully functional vascularized bone in-vitro as they can form all specialized cell types constituting the human bone, including its vasculature [195], ESCs research also raises ethical and political controversies due to their derivation from early human embryos [196]. For these reasons, the use of iPSCs obtained by reprogramming of adult somatic cells, thus avoiding the ethical problems inherent in ESC research, has gained significant attention.

Current models of biomimetic, engineered vascularized bone, fabricated by culturing human MSCs on 3D scaffolds resembling the matrix of native bone [197], require a vascular compartment created using other cell sources. Various sources of stem cells, such as ESCs, MSCs, and iPSCs, have been identified as potential candidates for vascular engineering [198–202]. Endothelial cells have also been successfully differentiated from amniotic fluid stem cells [203, 204]. The choice of vascular precursor cells is crucial for functional production of a proper vasculature within the in-vitro bone model. Mature ECs have been traditionally used to stimulate angiogenesis [205], among them HUVECs, which represent one of the most commonly employed cells in vascularized bone engineering. These cells naturally form vessel-like structures when cultured in hydrogels and, most importantly, they were demonstrated to enhance MSC osteogenesis in-vitro [86, 206–208]. Another cell type that has gained attention are the EPCs, which were found to be 10 times more proliferative than HUVECs [209] and have been recently used, also in combination with MSCs, to improve vasculogenesis in-vivo [210–213] and in tissue engineering applications [214–216]. Blood vessel development was also achieved by employing iPSC-derived ECs [217].

As mentioned earlier, active osteoclasts are also a crucial component of in-vitro bone modeling; however, the exact optimal cell source for osteoclasts has yet to be defined. There are several cell types that can be differentiated into osteoclast-like cells, such as bone marrow and peripheral blood mononuclear cells [109, 218], and human mononuclear leukocytes isolated from umbilical cord blood [219]. Osteoclasts can be directly isolated from native bone tissue [220]. RAW 264.7, a mouse leukemic monocyte–macrophage cell line, has been frequently used to study osteoclastogenesis in-vitro [221]. Primary monocytes can also be differentiated into osteoclast-like cells and used to study osteoclastogenesis in-vitro [222]. Furthermore, osteoclasts have been obtained by differentiating macrophages derived from human iPSCs [99].

**Fig. 4:**
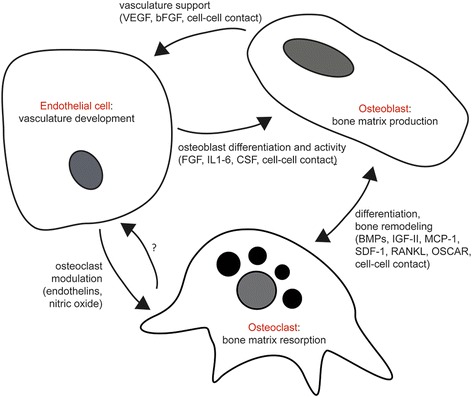
Primary cellular interactions between ECs, osteoblasts, and osteoclasts in production of vascularized bone. VEGF vascular endothelial growth factor, bFGF basic fibroblast growth factor, IL interleukin, CSF colony-stimulating factor, BMP bone morphogenetic protein, IGF insulin-like growth factor, MCP-1 monocyte chemoattractant protein-1, SDF stromal cell-derived factor-1, RANKL receptor activator of nuclear factor kappa-Β ligand, OSCAR osteoclast-associated receptor

These well-established, strong relationships among the constituent cell types, taken together, underscore the important and fundamental requirement of these cells in the success for development of vascularized bone in-vitro and the production of a veritable bone model (Fig. 4).

### Culture media

Based on the cells selected for the development of a vascularized bone construct, the choice of culture media and seeding methodology has to be carefully considered to avoid negative, undesired effects. Different medium formulations and supplements have been identified for optimal osteogenic differentiation of stem cells. Dexamethasone, β-glycerophosphate, ascorbic acid, 1,25-dihydroxyvitamin D_3_, and BMP-2 have significant osteogenic inductive activity on MSCs by influencing different aspects of their biology, such as activation of specific genes (osteopontin, core binding factor α1, alkaline phosphatase, osteocalcin) as well as signaling pathways like Wnt [184]. Regarding the culture of endothelial progenitors, VEGF, basic fibroblast growth factor (bFGF), and epidermal growth factor (EGF) are usually employed to stimulate endothelial progenitor expansion and differentiation/angiogenesis [223]. The use of individual differentiation medium, endothelial or osseous, generally yields satisfactory vasculature and mineralized matrix formation, albeit separately [224]. However, given the need for simultaneous development of bone and vasculature, a coculture system must be set up, and investigators have used either a combination of the two differentiation media [88, 206, 225–227], a single type of medium [88, 228], or osteogenic medium with some vascular growth factors [229]. In the case of a coculture system, while different cell types can be seeded simultaneously to avoid uneven distribution inside the construct and to allow communication between them, the choice of culture media is very important to assure cosurvival and differentiation. In another approach, different cell types can be expanded and differentiated separately and then seeded together, thus avoiding the media problem; however, some critical cell–cell interactions will be missing and, as discussed earlier, the generation of a veritable in-vitro model, which requires recapitulation of the interaction between bone cells and vasculature, may thus be compromised. Another parameter that needs to be considered is the cell ratio. Signaling between osteogenic and vascular precursors, as well as their adult counterparts in the mature organ, directs their functional integration (see Fig. 4). Altering the balance of this crosstalk compromises the engineering of the vascularized bone tissue. Investigators have studied varying combinations between osteogenic and vascular precursors, ranging from a 1:1 mix to unbalanced ratios in favor of osteogenic or endothelial precursors. Although a positive trend can be identified in the use of a 1:1 ratio, the results are also heterogeneous and an ideal ratio cannot be defined a priori [230]. Table 3 summarizes the main media and cell ratios that have been used in the engineering of vascularized bone and their major benefits and drawbacks.

**Table 3:** Main strategies of coculturing osteogenic and vascular precursors for vascularized bone engineering

Medium composition	Cell ratio (O:V)	Seeding methodology	
Osteogenic/vascular [88, 298]	1:1 [299]	Simultaneous seeding [229, 300, 301]	
Expansion/vascular [227]	1:4 [301]	Pros: technically simpler; even mix distribution within the construct; cell–cell crosstalk during differentiation	Cons: risk of suboptimal individual cell type viability and differentiation
Osteogenic [228]	2:1 [88]	Sequential seeding [89]	
Expansion [302]	4:1 [301]	Pros: optimal differentiation of the first seeded cells; cell–cell communication	Cons: uneven distribution of the two types of cells within the construct
Vascular [300]	5:1 [302]	Independent differentiation [88]	
Osteogenic with vascular growth factors [229]	8:1 [303]	Pros: optimal differentiation of each cell type in their respective medium	Cons: lack of cell–cell communication
	3:2 [300]		

*O* osteogenic, *V* vascular, *Expansion* expansion medium for osteogenic precursors

A recently published review by Liu et al. [83] covered different aspects of the medium combinations and seeding methodologies used in coculture systems for vascularized bone tissue engineering. However, due to the heterogeneity of the experimental parameters used in coculture studies, a clear trend is difficult to establish.

Considering the tight interactions between osteogenic and vascular stem cells during development, and the functional integration between bone and blood vessels in the mature organ, the development of veritable in-vitro models of this organ should comprise both types of cells cultured in a mixed medium in order to not miss the key interplay between osseous and vascular precursors/mature cells.

### Bioreactors

**Fig. 5:**
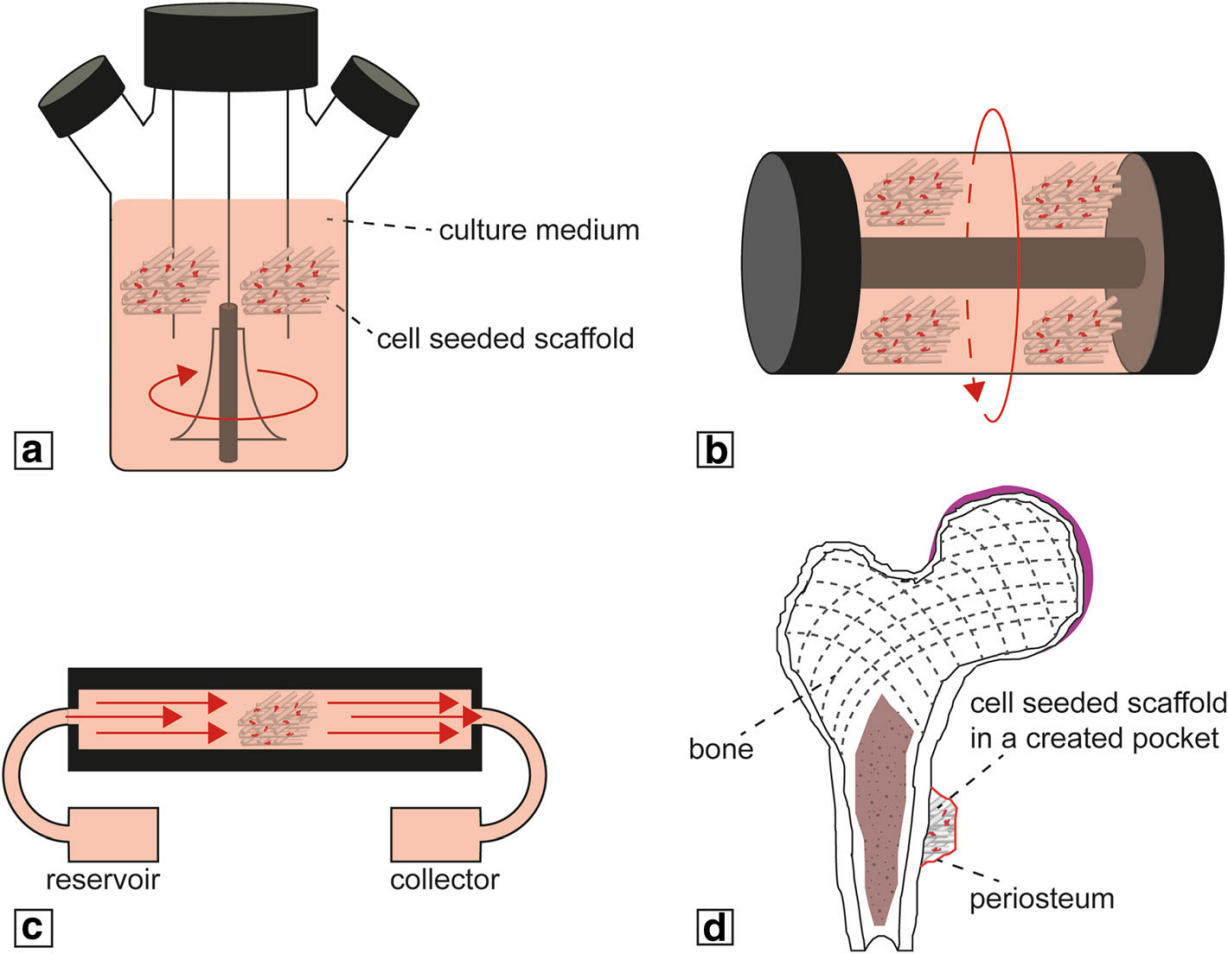
Schematic of main bioreactors used for production of 3D constructs for TE applications: **a** spinner flask, **b** rotating wall vessel, **c** perfusion, and **d** in-vivo bioreactor

Bioreactors are generally defined as any device that is able to dynamically sustain biological and/or biochemical processes under precisely monitored and controlled experimental and operating conditions (e.g., pH, temperature, mechanical stimuli, time, nutrient supply, and waste removal) [231]. Different types of experimental setups have been developed to optimize cell seeding and mass transport, such as spinner flasks (Fig. 5a), rotating wall vessels (RWVs) (Fig. 5b), and perfusion bioreactors (Fig. 5c). Spinner flasks and RWVs minimize the nutrient gradient and metabolite concentration around the construct, while perfusion bioreactors directly perfuse media inside the scaffold, thus assuring mass transport within the porosity [232]. Using optimal material–scaffold–cell systems, coupled with the higher control over experimental parameters, has made bioreactors ideal means for the development of 3D tissues in-vitro. This is particularly true for the engineering of biological interfaces or complex tissues, like the osteochondral junction and vascularized bone. Bioreactors, in combination with MSCs, have in fact been used for the recreation of the osteochondral tissue interface, as reported in a number of studies [121, 233, 234]. In particular, culturing in flow perfusion bioreactors has been shown to upregulate expression of osteoblastic markers [235–237], and these bioreactors have been employed for the production of engineered bone [238–240]. To improve the amount and quality of bone beyond what has been produced by an in-vitro process, various studies have reported the use of “in-vivo bioreactor” systems to produce a clinically relevant amount of vascularized bone (Fig. 5d). These approaches were based on the body’s healing mechanism that supports the formation of neotissue in different kinds of scaffolds, ranging from calcium-alginate gel, to β-TCP, to natural bovine bone mineral-coated titanium meshes implanted subperiosteally in-vivo [241–243]. The engineered bone produced with this approach could be harvested from the “living bioreactor” host and subsequently transplanted successfully at a bone defect site. While in-vivo engineering surely ensures production of enhanced vascularized bone, high-throughput assays require a large number of identical samples that cannot be performed by in-vivo systems, thus necessitating the development of in-vitro platforms of vascularized bone engineering. In addition to improving in-vitro osteogenic differentiation of MSCs in the presence of ECs, several studies have reported better matrix mineralization by osteogenic MSCs cultured under dynamic conditions rather than static ones [237, 244–246]. However, the combination of these two strategies using a flow perfusion bioreactor did not significantly enhance osteogenic differentiation of MSCs in the presence of ECs [247, 248], likely because shear stress affects the function of ECs [249–251]. However, another approach was used by Nishi et al. [252], who cultured MSCs and ECs on a porous poly(lactic acid) scaffold in a rotating wall vessel bioreactor. The flow environment created by the RWV bioreactor, coupled with the interaction between the cell types, enhanced the distribution and differentiation of cells in the scaffold. To date, there are only a limited number of studies using a bioreactor in combination with bone and vascular progenitor cells, due to the technical and biological challenges of codifferentiating the two cell types together. The successful construction of a physiologically compatible bioreactor for engineered vascularized bone, to achieve fast and easy visualization of the target cells and their interactions, must take into account a number of design features, including noninvasive optical access, automation, uniform cell seeding, overall chamber dimensions, checkpoint markers, and mechanical stimuli [253, 254].

In utilizing engineered vascularized bone models in-vitro, instead of harvesting tissue samples and analyzing them at designated time points, the nondestructive tracking of cells and new matrix formation should result in a reduction of resources and permit real-time understanding of the processes. One example is represented by the work reported by Bersini et al. [255], who developed a biological 3D in-vitro microfluidic model to study breast cancer metastasis to bone. This microfluidics platform is based on hydrogel-containing chambers placed between surface-accessible microchannels, allowing high-resolution real-time imaging of single-cell behavior, cell–cell communication, cell–matrix interactions, and cell population dynamics [256]. Another useful high-throughput platform was developed by Moya et al. [257] to study vasculature in real time. They produced an in-vitro 3D metabolically active stroma (~ 1 mm^3^ volume) containing a living and dynamically perfused human capillary network, by combining human endothelial colony forming cell-derived ECs (ECFC-ECs) and a polydimethylsiloxane (PDMS) micromold. Although diffusion of oxygen was demonstrated to not be a limiting step in-vitro, thus allowing the development of tissue constructs in the order of centimeters in thickness [258], the major technical problem is nutrient diffusion. The density of the ECM plays an important role in the diffusion of nutrients and the morphogenesis of capillaries [259], and this is particularly true for mineralized bone matrix. In addition, real-time visualization of the process occurring inside the bone matrix is also limited by the opacity of the bone matrix itself, thus requiring the development of specific bioreactor or in-vitro models to overcome this limitation. X-ray microcomputed tomography (μCT) has been extensively used in the field of bone tissue engineering due to its ability to provide rapid, nondestructive 3D images of bone and bone scaffold microstructure at 1–50 μm resolution [260, 261]. Various groups have developed μCT-compatible bioreactors [262, 263], utilizing low radio-opacity materials, such as polysulfone, with dimensions matched to those of standard μCT chambers. Another requirement is that such systems should allow the study of the vasculature within in-vitro vascularized bone, which, however, is complicated by the 3D nature and limited access to the internal microvasculature due to the loss of optical access. While the analysis of thin sections of engineered vascularized bone could address this issue, it would obviate the purpose of recapitulating the 3D in-vivo structure. A more viable solution is the use of contrast-enhanced μCT for the investigation of microvasculature [264], which was previously studied in soft tissues, such as the kidney, heart, and liver [265–267]. This method is based on the injection of a radio-opaque contrast agent within the vasculature prior to imaging. However, again, the opacity and density of the bone matrix put some limitations on visualization of the vessel microstructure when this is performed in a bone model. It is thus not surprising that few studies have been published on the analysis of bone microvasculature using μCT [268, 269], illustrating the future potential of contrast-enhanced μCT for the evaluation of bone neovascularization in tissue-engineered constructs.

## Perspectives and future directions

The definition of the parameters that must be accommodated in the design of in-vitro models of vascularized bone represents a challenging task for researchers. The complex interactions between blood vessels and bone have limited our ability to develop veritable in-vitro constructs of physiological systems representing human bone. First, from a biological point of view, the codifferentiation of endothelial precursors and osteogenic stem cells is hindered by the different mechanisms and signals to which these cells respond when recapitulating in-vivo development. As shown in numerous studies, a compromise between endothelial and osteogenic signals/cues must be considered, in order to minimize undesired differentiation of the cell types. On the other hand, predifferentiation of vasculature or bone cells does not permit the critical communications between the two physiological systems during development or repair, thus compromising the morphogenesis of the actual structure of the tissue complex and in a manner similar to native bone. This aspect is of high relevance when studying the effects of drugs and/or toxicants on the structure and development of bone, as well as screening for potential treatments. In other words, the engineered vascularized bone model must present advantages over the majority of studies that have been performed in-vitro on 2D cultures of separate endothelial and osteogenic cells or cocultures, and through in-vivo animal models [270, 271]. From an engineering point of view, the design of a system which could maintain the vitality and differentiation of different types of cells for long-term culture is also a significant challenge. Furthermore, experimental parameters must be controlled (i.e., system variables must be clearly defined) and, as described earlier, the system must be accessible for real-time imaging and evaluation. Bioreactors have partially solved the need to have in-vitro systems which could mimic the full physiological structure of bone and, at the same time, have allowed analysis of the constructs.

High-throughput screening of drugs or toxicant relies on a minimal system defined by precise parameters that can be stimulated by specific stimuli, such as mechanical, biochemical, genetic, and so forth. To address this, our group has developed a triphasic model composed of blood vessels, bone, and cartilage to study the interaction between these three different tissues [128]. Our 3D-printed microphysiological system, in combination with stem cells, allows for the codifferentiation of the three different tissues, and is also responsive to the introduction of different stimuli, including stress-inducing factors, such as IL-1β for the study of osteoarthritis, female hormones like estrogens for the study of osteoporosis [272], or teratogens to study their effect on skeletal development. The capabilities of additive manufacturing have enabled us to produce customized scaffolds and bioreactors to meet the requirements of vascularized bone engineering, coupled with a high-throughput platform for drug screening and toxicology assessment. In the future, bioprinting could represent a suitable on-demand platform for the versatile fabrication of specific cellular patterns at the micrometer scale for the production of vascularized bone, but also a broad range of other engineered tissues [166]. While still in its early stage, bioprinting can also benefit from the wide range of additive manufacturing techniques and bioreactor technologies to generate veritable in-vitro models of vascularized bone that could help understand the physiopathology of bone, and generate valuable treatment strategies. To become an economically viable resource, the production of in-vitro 3D models of vascularized bone should also consider the introduction of a certain level of automation, to reduce human intervention as well as product variability. An interesting and comprehensive review about this topic was recently published by Costa et al. [273], who reviewed the current automated tools and strategies for the production of bone substitutes.

## Conclusions

In addition to applications in regenerative medicine, tissue-engineered constructs could be used as in-vitro preclinical models of normal or pathological tissues for applications in drug screening and toxicity assessment. For effective high-throughput assays, minimal systems and accurate modeling of the structure/function of the studied human tissue or organ are required. Bone pathologies present significant disease burden, underscoring the need to develop more effective treatment strategies. The development of in-vitro high-throughput microphysiological models that faithfully recapitulate the physiopathology of bone is thus highly relevant, particularly given the cost and intrinsic genetic difference of animal models. Different biological and engineering challenges must be overcome, including the right choice of stem cells, regulation of codifferentiation of vasculature and bone, control of system parameters and stimuli, access for real-time imaging, and functional evaluation. In the pursuit of this initiative, additive manufacturing techniques and bioreactor technologies have increased our ability to produce systems that integrate cells, scaffolds, and biological/environmental stimuli to create in-vitro models of native osseous tissue, and to study the processes regulating its biology.

## Abbreviations

EC: Endothelial cell
EPC: Endothelial progenitor cell
ESC: Embryonic stem cell
HUVEC: Human umbilical vein endothelial cell
iPSC: Induced pluripotent stem cell
MSC: Mesenchymal stem cell
μCT: Microcomputed tomography
